# Nitrogen *K*-Edge X-ray Absorption Spectra of Ammonium and Ammonia in Water Solution: Assessing the Performance of Polarizable Embedding Coupled Cluster Methods

**DOI:** 10.1021/acs.jpclett.1c02031

**Published:** 2021-09-09

**Authors:** Peter Reinholdt, Marta L. Vidal, Jacob Kongsted, Marcella Iannuzzi, Sonia Coriani, Michael Odelius

**Affiliations:** † Institut for Fysik, Kemi og Farmaci, Syddansk Universitet, DK-5230 Odense, Denmark; ‡ DTU Chemistry, Technical University of Denmark, DK-2800 Kongens Lyngby, Denmark; § Physical Chemistry Institute, University of Zürich, 8057 Zürich, Switzerland; ∥ Department of Physics, AlbaNova University Center, Stockholm University, SE-106 91 Stockholm, Sweden

## Abstract

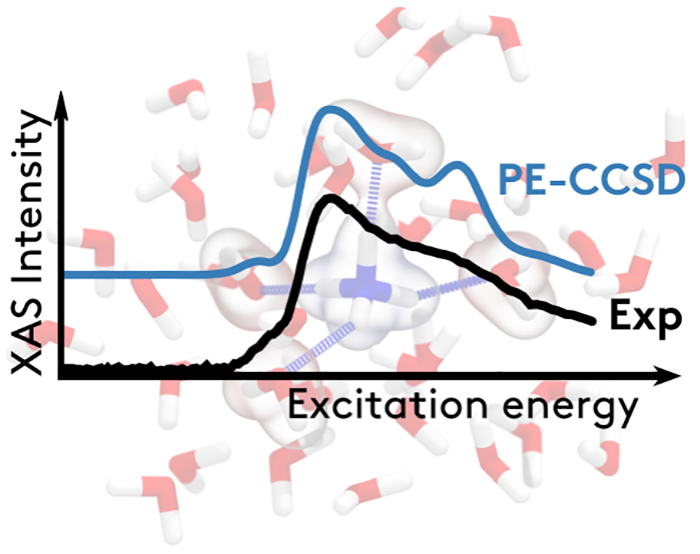

The recent development of liquid jet and liquid leaf sample delivery systems allows for accurate measurements of soft X-ray absorption spectra in transmission mode of solutes in a liquid environment. As this type of measurement becomes increasingly accessible, there is a strong need for reliable theoretical methods for assisting in the interpretation of the experimental data. Coupled cluster methods have been extensively developed over the past decade to simulate X-ray absorption in the gas phase. Their performance for solvated species, on the contrary, remains largely unexplored. Here, we investigate the current state of the art of coupled cluster modeling of nitrogen *K*-edge X-ray absorption of aqueous ammonia and ammonium based on quantum mechanics/molecular mechanics, where both the level of coupled cluster calculations and polarizable embedding are scrutinized. The results are compared to existing experimental data as well as simulations based on transition potential density functional theory.

Insight into the hydration of biomolecules and simple ions on a molecular level is important for understanding, e.g., the mechanisms of enzymatic reactions and of dissolution of minerals. Numerous powerful experimental probes have been developed for the study of hydration structure and hydrogen bond dynamics. Knowledge of how the electronic structure of solutions is affected by hydration interactions has been gained through photoelectron spectroscopy and X-ray spectroscopy.^1−4^ X-ray absorption (XA) spectroscopy^2,5,6^ is very sensitive to hydration, in particular hydrogen bonding, because the underlying core-excited states often involve extended unoccupied orbitals that are very sensitive to slight geometric changes. To date, most XA studies of aqueous solutions and protic solvents have been performed at synchrotron facilities, but the recent development of high harmonic generation is enabling XA measurements using laboratory-based sources.^7^ Together with the improvement in sample delivery and measurement geometry using, e.g., liquid flatjets,^8^ accurate XA spectra can be acquired.

Because X-ray spectroscopy reaches the core levels, the photon energy of the incident X-rays can be tuned to specific element edges to give element selectivity, and the local electronic structure of a solute can be probed in a complex environment. Hence, XA spectroscopy has been used to study the hydration of amino acids and dipeptides^9^ and the influence of salinity on the hydration of proteins.^10^ It is a local probe that is primarily determined by covalent bonding,^5^ but XA spectra are still strongly influenced by hydration as seen in systematic studies of the pH dependence of nitrogen *K*-edge XA spectroscopy of aqueous amino acids and alkylamine in protic solvents.^9,11,12^

Hydrogen bonding interactions in aqueous ammonia (NH_3_) and ammonium ion (NH_4_^+^), two key amine compounds in aqueous solution, have been the subject of extensive theoretical and experimental investigations in recent years.^13−16^ A quantitative assessment in terms of electronic structure, solvation structure, and dynamics was obtained by combining local soft X-ray and vibrational infrared spectroscopic results with ab initio molecular dynamics simulations based on density functional theory (DFT).^13^ The ammonia molecule was shown to have a strong asymmetry in hydrogen bonding to the solvent, with weakly donating hydrogen bonds and a very strong accepting hydrogen bond, whereas the ammonium ion is involved in strong hydrogen bond donation. Theoretical modeling is a fruitful and necessary complement for a trustworthy interpretation of X-ray spectra, but shortcomings of the employed DFT methods for spectrum simulations are apparent.^13^ Given the improved experimental spectra of solutions, there is a need to develop accurate methods for XA spectrum calculations of explicitly solvated solutes, and it is important to investigate how to best balance the required level of quantum chemistry with the environment.^17^

In this paper, we will use the previously published experimental XA spectra^13^ of NH_3_(aq) and NH_4_^+^(aq) as a test case to assess the performance of the coupled cluster linear response^18^ methods CC2 (coupled cluster singles and approximate doubles^19^) and CCSD (coupled cluster singles and doubles^20,21^). In particular, we will investigate whether the shortcomings of the transition potential DFT (TP-DFT) methods in the description of the post-edge region of the nitrogen *K*-edge spectra of the solutes can be overcome.^13^

CC2 and CCSD calculations of the XA spectral parameters were performed using Dalton^22^ by applying the core–valence separation during the solution of the coupled cluster eigenvalue equations^23^ to selectively target the core-excited states. The polarizable embedding (PE)^24,25^ coupled cluster^26^ framework was used to account for solvent effects. We considered 194 sample structures for both NH_3_ and NH_4_^+^ in aqueous solution, constructed from the same configurations of previous ab initio molecular dynamics (AIMD) simulations selected for spectral calculations in ref (13). Each snapshot originally contained 63 water molecules and one solute molecule under periodic boundary conditions. From each of these AIMD samples, we built our model structures by extracting the solute molecule (NH_3_/NH_4_^+^) and four neighboring H_2_O molecules and placed them into the quantum mechanical (QM) region. The remaining water molecules (H and O atoms thereof) were used as reference points for the PE description of the molecular mechanics (MM) environment. A model of the chosen QM/MM space is shown in Figure 1.

**Figure 1:**
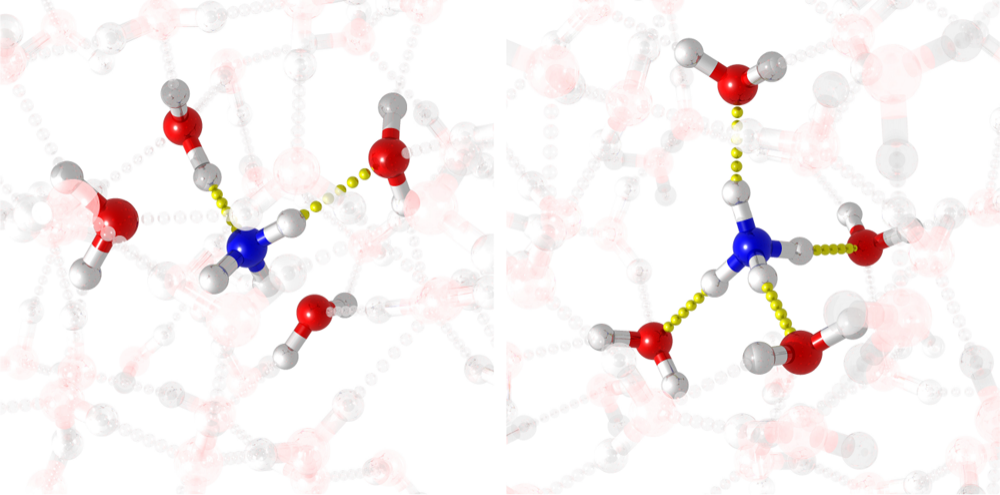
Model systems for aqueous NH_3_ and NH_4_^+^. The QM region is represented in bright colors, and the classical MM region with transparency. Color coding is employed for elements: blue for nitrogen atoms, red for oxygen atoms, and white for hydrogen atoms.

The number of water molecules in the QM region is kept the same in all configurations and was chosen on the basis of the number of hydrogen bonds between the solute and the solvent. Structure-specific PE parameters were derived with the LoProp^27,28^ method based on CAMB3LYP^29^/aug-cc-pVTZ calculations on the individual water molecules. The atom-centered parameters consisted of multipoles (up to and including quadrupoles) and anisotropic polarizabilities. The calculation of embedding parameters was automated using the PyFraME^30^ python package. As discussed below, several tests were run to identify any potential problems and/or limitations in our choice of PE parameters. For a tutorial on the use of the PE framework for the calculation of molecular properties of, e.g., solvated molecules, we refer to ref (31).

All core excitation energies and corresponding transition strengths and/or oscillator strengths were computed for each sample structure up to 425 eV (30 excited states). The nitrogen *K*-edge XA spectrum of each sample was generated by applying a Gaussian broadening with a full width at half-maximum (fwhm) value of 0.4 eV. Averaged theoretical XA spectra of aqueous NH_3_ and NH_4_^+^ were finally computed by sampling all of the configurations of each species. The core-ionization potentials of all sampled configurations were also computed, and a corresponding averaged value was then determined and related to the transitions in the XA spectra.

Preliminary PE calculations were carried out on two arbitrary snapshots of the NH_3_ and NH_4_^+^ systems with the aim of determining the basis set requirements. Use of the same basis set on the solute and solvent molecules in the QM region (either 6-311++G** or 6-311G**) or combinations with different basis sets on solute and solvent atoms (6-311++G**/6-311G** and 6-311++G**/6-31G**), as well as adding effective core potentials to avoid spurious electron spill-out effects,^32^ were tested. While the mixed basis set approach was attractive from a computational cost point of view, we observed very sharp spectral features in the 409–410 region of the spectra when adopting the 6-311++G** basis set on ammonia/ammonium and smaller basis sets (6-31G** and 6-311G**) on water (see Figure 2). These features were significantly “smeared out” when using the same basis set on both ammonia/ammonium and the QM water molecules. Ultimately, we therefore opted for the more flexible 6-311++G** basis set for all QM atoms. We also investigated the effect of using a more flexible description of the core orbitals. Following recent prescriptions,^33^ we uncontracted the 1s functions alone, as well as all functions in the Pople set of nitrogen. The results for a selected structure are shown in Figure S3. The increased flexibility of the basis set when uncontracting the inner functions resulted in a rigid shift of the whole spectrum, without any additional spectral features. The fully uncontracted set gave basically the same results as the regular, contracted, basis. For the sake of computational convenience, we opted for the regular, contracted set.

**Figure 2:**
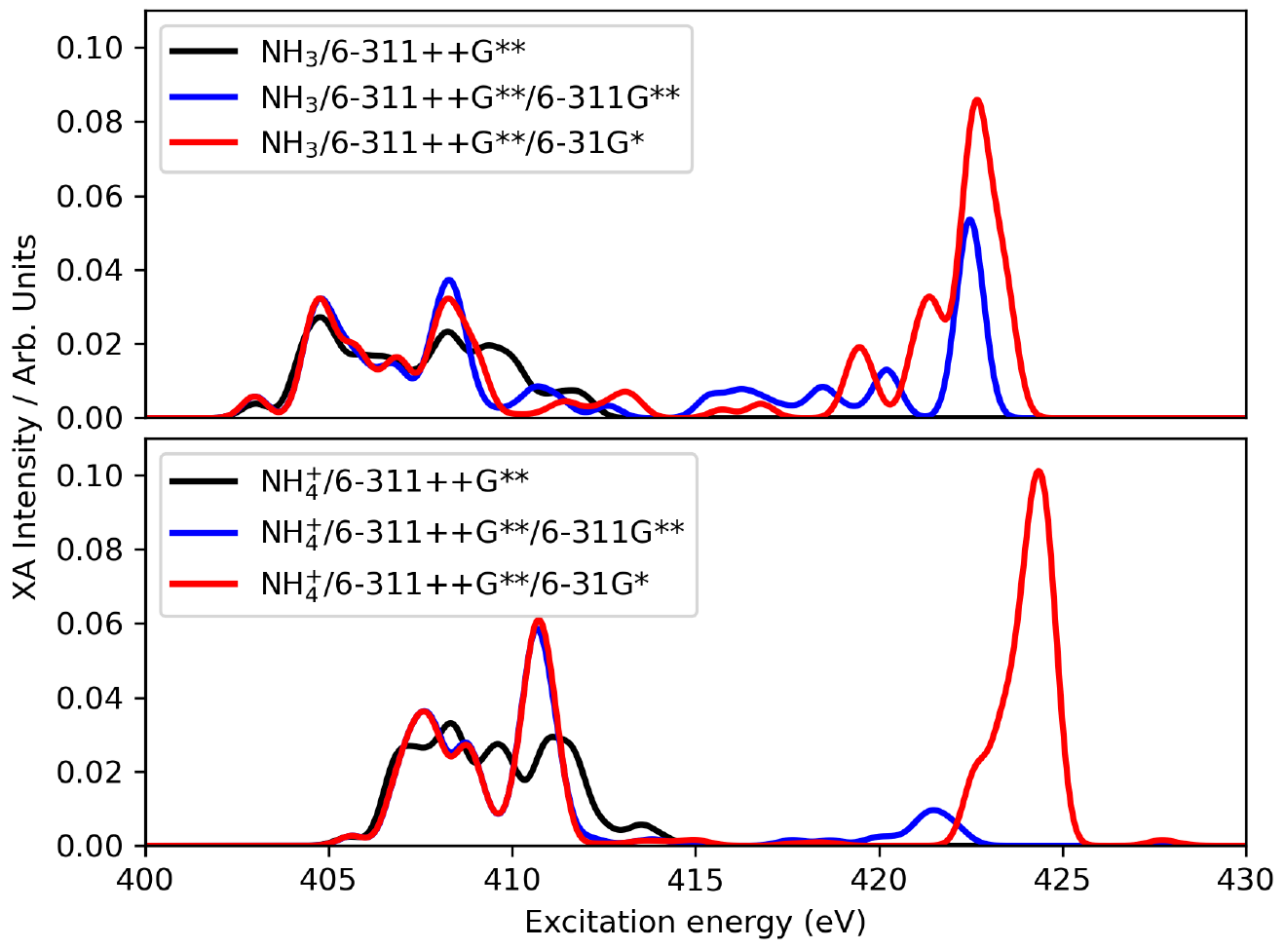
PE-CCSD basis set investigation. The shown spectra are averages over two snapshots (“step 3000” and “step 5000”). Thirty excited states were considered in all cases.

The inclusion of solvation effects was analyzed at different levels. With reference to Figures S1 and S2, we first assessed the importance of including a PE description of solvation, by comparing the spectra obtained for four representative snapshots of both species surrounded by four quantum-chemical water molecules with and without the PE environment.

The results clearly show that including a PE description of the solvation environment is important. We then further analyzed the effect of different choices of the PE parameters. Among other aspects, e.g., charges parametrization (Figure S4), we investigated the differences between using isotropic and anisotropic polarizabilities in the PE (see Figure S5). Modest, yet noticeable, differences were observed; therefore, anisotropic polarizabilities were subsequently used as they were considered more accurate. The importance of effective external field (EEF) effects^34^ was examined next (see Figure S6). As the differences were extremely modest, the EFF effects were neglected in the remaining calculations.

An additional aspect was considered, which relates to the description of the two clusters, namely, the effect of the use of a larger replicated region. In Figure S7, we show the CCSD spectra obtained, again for two selected structures, adopting an inner polarizable region of 12 Å polarizable LoProp water molecules both alone and together with an outer nonpolarizable region up to 25 Å of SPC water molecules to mimic bulk water effects. Clearly, the inclusion of more water at the MM level does not have a huge effect.

Having chosen the parametrization of the polarizable embedding, we then carried out XA spectral calculations at the CC2 and CCSD levels of theory for two randomly selected snapshot structures. The spectra are shown in Figure S8. Already on the basis of these two structures, we noticed quite significant differences between the CC2 and CCSD predictions, especially for the protonated species, which we considered a preliminary indication that the CC2 method might not be sufficiently accurate. Nonetheless, we opted to carry out the CC2 calculations on the complete set of available snapshots. Figure 3 presents the final averaged XA spectra of the two methods.

**Figure 3:**
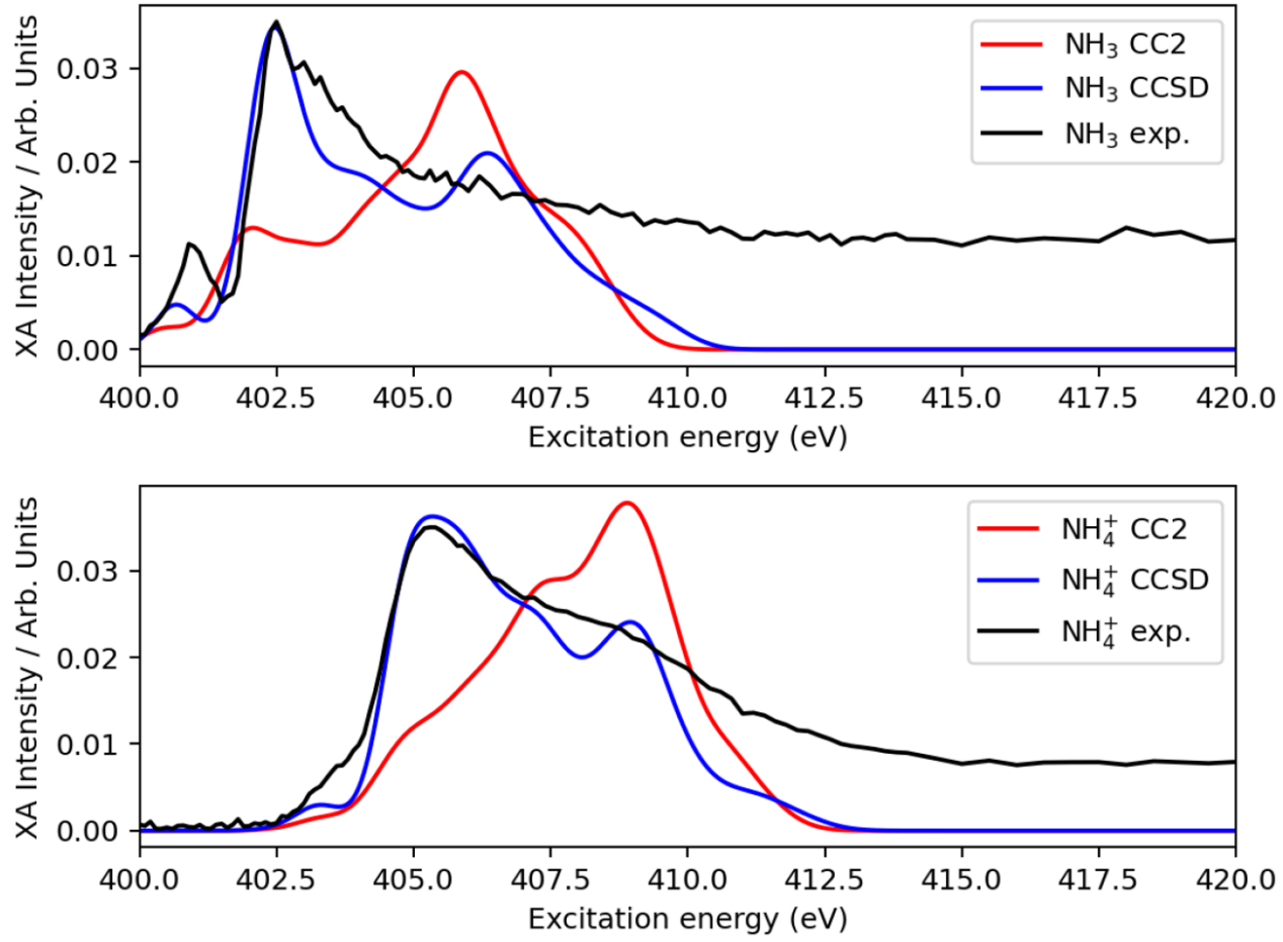
Averaged X-ray absorption spectra of ammonia and ammonium in water using PE-CCSD (blue) and PE-CC2 (red) and the 6-311++G** basis set. Experimental results from ref (13) are colored black. The CC spectra are shifted to align with the experimental spectra.

From this analysis, and having the experimental spectra in mind,^13^ we find that CC2 clearly fails in describing the nitrogen *K*-edge XA spectra of aqueous ammonia and ammonium, not only in the post-edge region but also in the pre-edge region. We attribute this to an inability of CC2 to properly account for relaxation effects. Orbital relaxation is one of the dominant effects in core spectroscopy. In propagator methods like CC response, this orbital relaxation is accounted for via electron correlation. CC2 is derived from CCSD based on a perturbational analysis of the cluster amplitude equations.^19^ The singles equations are retained as such, whereas the doubles equation is approximated to first order, the lowest nonvanishing order in perturbation theory.^19^ The CC2 Jacobian, from which excitation energies are obtained, is diagonal in the doubles–doubles block, with the HF orbital energy differences as the diagonal elements. It is thus reasonable to conclude that the evident failure of CC2 in our case is connected to the simplifications introduced in CC2 compared to CCSD, i.e., in the way the double amplitudes are approximated and how these approximations affect the calculation of excitation energies and transition moments. In CC2, single excitations are correct to second order like in CCSD; however, the latter completely includes all singles and doubles terms, whereas CC2 does not. Transition moments are correct to first order, versus second order of CCSD. Double excited states are not described in CC2. In the gas phase, CC2 has previously been shown to yield compressed XA spectra.^35−37^

Having established the unreliability of CC2, we then focused on the performance of CCSD, both with respect to experiment and with respect to the results of previous calculations based on TP-DFT.^13^ In Figures S9 and S10, we thus compared the XA spectra obtained, at the PE-CCSD/6-311++G** level, for four different snapshot structures of each species, together with their total averaged spectra and the experimental spectra. Despite the differences between the spectra of the four snapshots, one clear picture emerges. In the case of ammonium, CCSD yields significant intensity in the post-edge region, in agreement with the experimental profile.

Figure 4 shows the superposition of the CCSD spectra of all snapshots, together with their final averages. The vertical shadowed areas are the superposition of the ionization energies of all individual snapshots, whereas the vertical line in each plot is their average. Note that the spectra have been shifted by −1.95 eV (to align the main peaks with the experimental ones).

**Figure 4:**
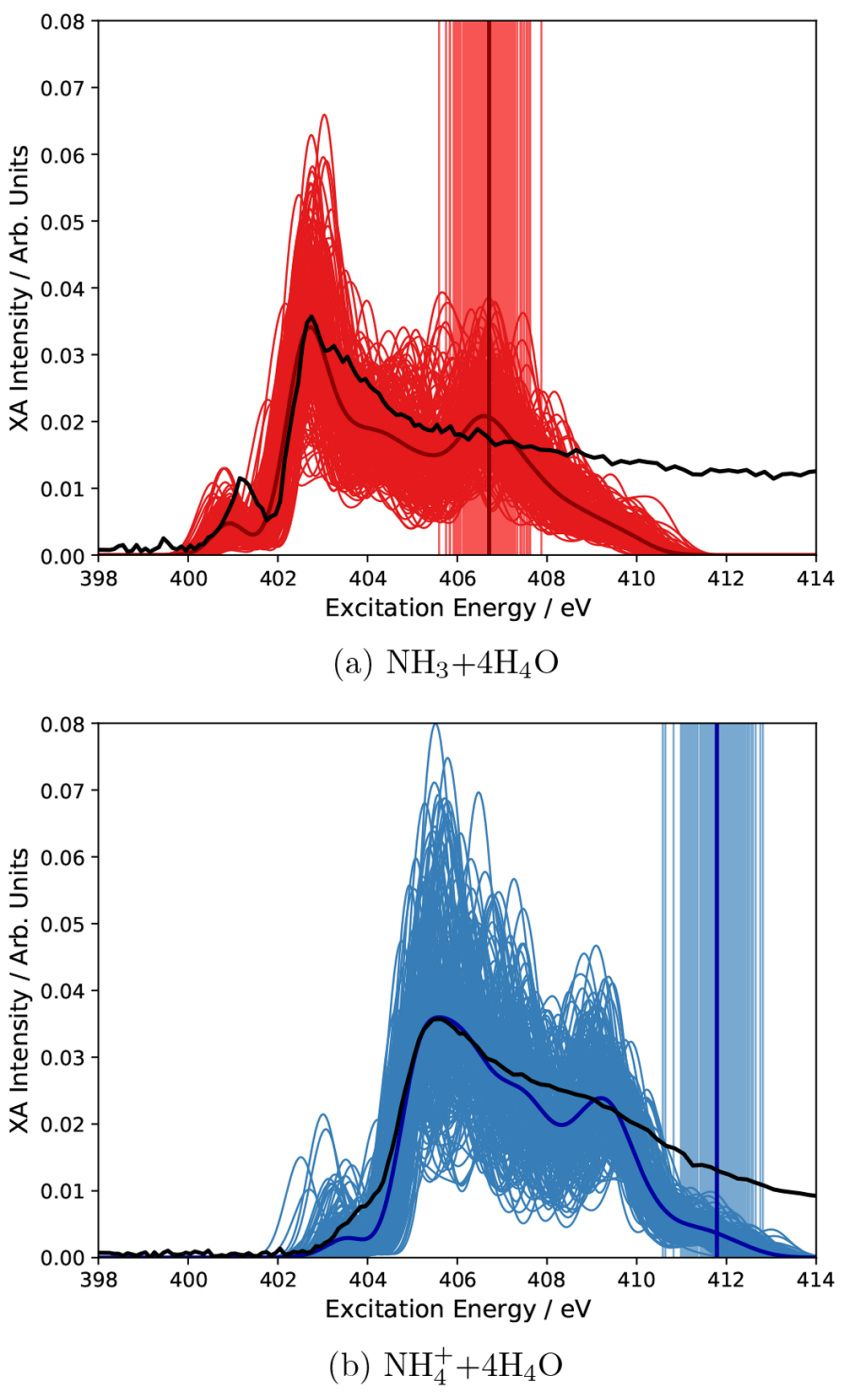
PE-CCSD/6-311++G** X-ray absorption spectra of ammonia and ammonium in water. The spectra of all snapshots are shown, together with their averages (thicker lines). All spectra have been shifted by −1.95 eV, to align with the experimental results from ref (13), colored black. The vertical colored thin lines are the ionization energies of the individual snapshots, and the vertical thick line is the average ionization energy.

In the case of ammonium, the intensity in the post-edge region is well below the ionization limit. We believe this is a strong indication that the observed post-edge intensity is not due to artifacts in the description of the continuum. This contrasts the spectral feature at ∼407 eV for ammonia, which is not present in the experimental spectrum, and overlaps with the ionization region. We attribute this band to artifacts due to the discretized representation of the continuum.

Our CCSD result for ammonium is at variance with what has been previously observed at the level of TP-DFT,^13^ as illustrated in the comparative plot in Figure 5.

**Figure 5:**
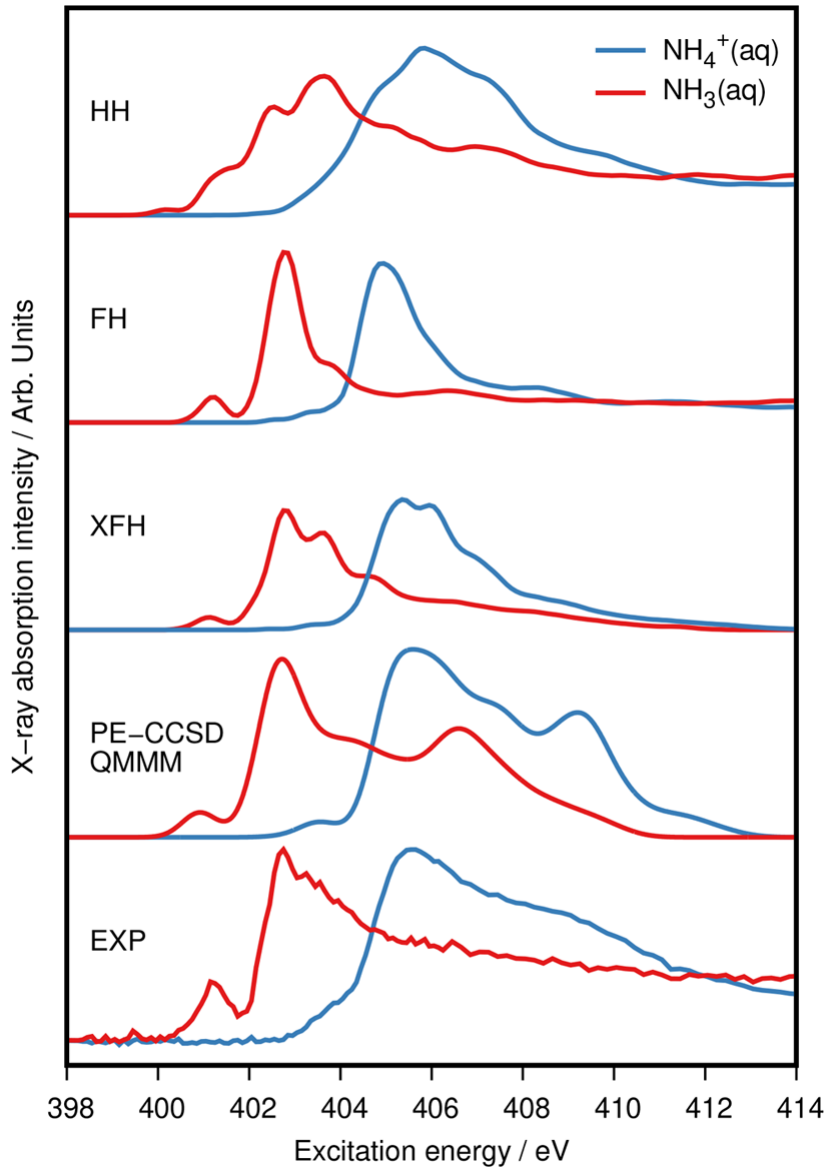
Averaged spectra of NH_3_ and NH_4_^+^ in water solutions for different computational methods vs experiment (label EXP) from ref (13). HH indicates the half-core-hole, FH the full-core-hole, and XFH the full-core-hole excited TP-DFT results from ref (13). PE-CCSD QMMM are the results obtained in this study. See the text for details.

As shown in Figure 5, the experimental XA spectrum of aqueous NH_3_ is characterized by a pre-edge peak at 401.2 eV, a sharp main band at 402.8 eV, and a tail due to post-edge transitions between 403 and 414 eV. In the XA spectrum of NH_4_^+^, the main peak is centered at 405.7 eV, preceded by a small shoulder at 403–404 eV, corresponding to the pre-edge peak. A rather pronounced post-edge feature is present between 407 and 411 eV, with peak intensity at around 409 eV.^13^

PE-CCSD is evidently the only method in Figure 5 capable of satisfactorily reproducing the entire experimental spectrum of both systems. All three transition potential approaches considered in ref (13), namely, the half-core-hole (HH) transition potential method, the full-core-hole (FH) transition potential method, and the full-core-hole excited (XFH) approximation,^38,39^ yield spectra with a very rapid intensity decay after the sharp main-edge feature. We thus conclude that inclusion of double excitations (as well as solvent) in the wave function parametrization is important to account for the excitation and relaxation processes at play, not only in the pre- and main-edge regions but also in the post-edge region, as also indicated by the failure of the PE-CC2 approach.

As a final comment, we notice from the comparison of XA spectra obtained using different QM regions in the XFH QM/MM calculations in Figure S11 that limiting the QM region gives rise to finite size effects in the XA spectra with a feature appearing 2–3 eV above the main edge for both solutes. These features are smeared in the case of a larger QM region due to orbital mixing with the surrounding water molecules. However, the post-edge features in the PE-CCSD QM/MM calculations are higher in energy, and in the case of ammonium, they align nicely with the position of the experimental post-edge feature. Hence, we deem this to be a significant improvement in the CCSD treatment and not an artifact of the QM/MM approximation. It would be of interest to further validate our conclusions versus multilevel coupled cluster approaches for core excitations^40,41^ as well as other types of CC calculations in which the QM region can be extended to the second solvation shell.

In conclusion, we have explored the performance of coupled cluster CC2 and CCSD with a polarizable embedding for XA spectra in water solutions. While CC2 was found to be inadequate, we have shown very promising results for the XA spectra of aqueous NH_3_ and NH_4_^+^ at the PE-CCSD level. We establish a stable description with respect to the choice of basis set and level of the PE description. We hope our results will stimulate further investigations of XA spectra of molecular liquids and electrolyte solutions, which could give a more accurate interpretation of experimental data.

## Supporting Information Available

Effect of including polarizable embedding (PE), effect of increasing the basis set flexibility in the core region, choice of charges parametrization, isotropic versus anisotropic polarizabilities, effective external field (EEF) effects, bulk water effects, two snapshots of CC2 versus CCSD, XAS for four representative structures, effect of enlarging the QM/MM spaces for TP-DFT, and comparison of PE-CCSD with CPP-PE-TDDFT/CAM-B3LYP (PDF)
