# Supplementary material for: Nitrogen K-Edge X-ray Absorption Spectra of Ammonium and Ammonia in Water Solution: Assessing the Performance of Polarizable Embedding Coupled Cluster Methods
Source: J Phys Chem Lett. 2021 Sep 9;12(36):8865–71. doi: 10.1021/acs.jpclett.1c02031 (PMC8450933; doi:10.1021/acs.jpclett.1c02031)
Supplement: Supplementary file 1 — jz1c02031_si_001.pdf [file jz1c02031_si_001.pdf]

Nitrogen K-edge X-ray absorption Spectra of Ammonium and Ammonia in Water Solution: Assessing the Performance of Polarizable Embedding Coupled Cluster Methods. Supplementary Information.

Peter Reinholdt,[†,‖] Marta L. Vidal,[‡,‖] Jacob Kongsted,[†] Marcella Iannuzzi,[¶] Sonia Coriani,[*,‡] and Michael Odelius[*,§]

†*Institut for Fysik, Kemi og Farmaci, Syddansk Universitet, Odense, Denmark*

‡*Department of Chemistry, Technical University of Denmark, DK-2800 Kongens Lyngby, Denmark*

¶*Physical Chemistry Institute, University of Zürich, 8057 Zürich, Switzerland*

§*FYSIKUM, Albanova, Stockholm University, S-106 91 Stockholm, Sweden*

‖*These authors contributed equally*

E-mail: soco@kemi.dtu.dk; odelius@fysik.su.se

Effect of Including the Polarizable Embedding (PE)

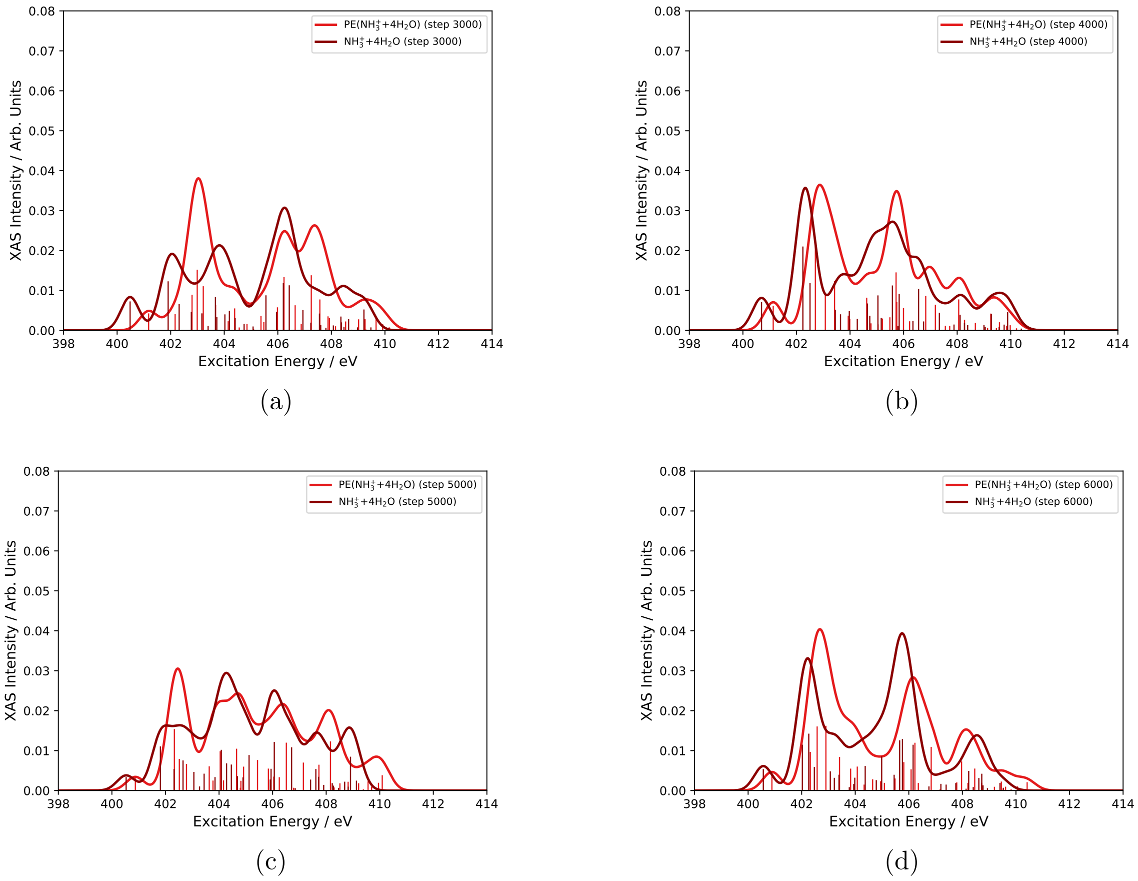

Figure S1: X-ray absorption spectra of ammonia at selected snapshots (3000-6000) surrounded by 4 water molecules with and without polarizable embedding (PE) at the CCSD/6-311++G** level of theory. The spectra have been shifted by −1.95 eV.

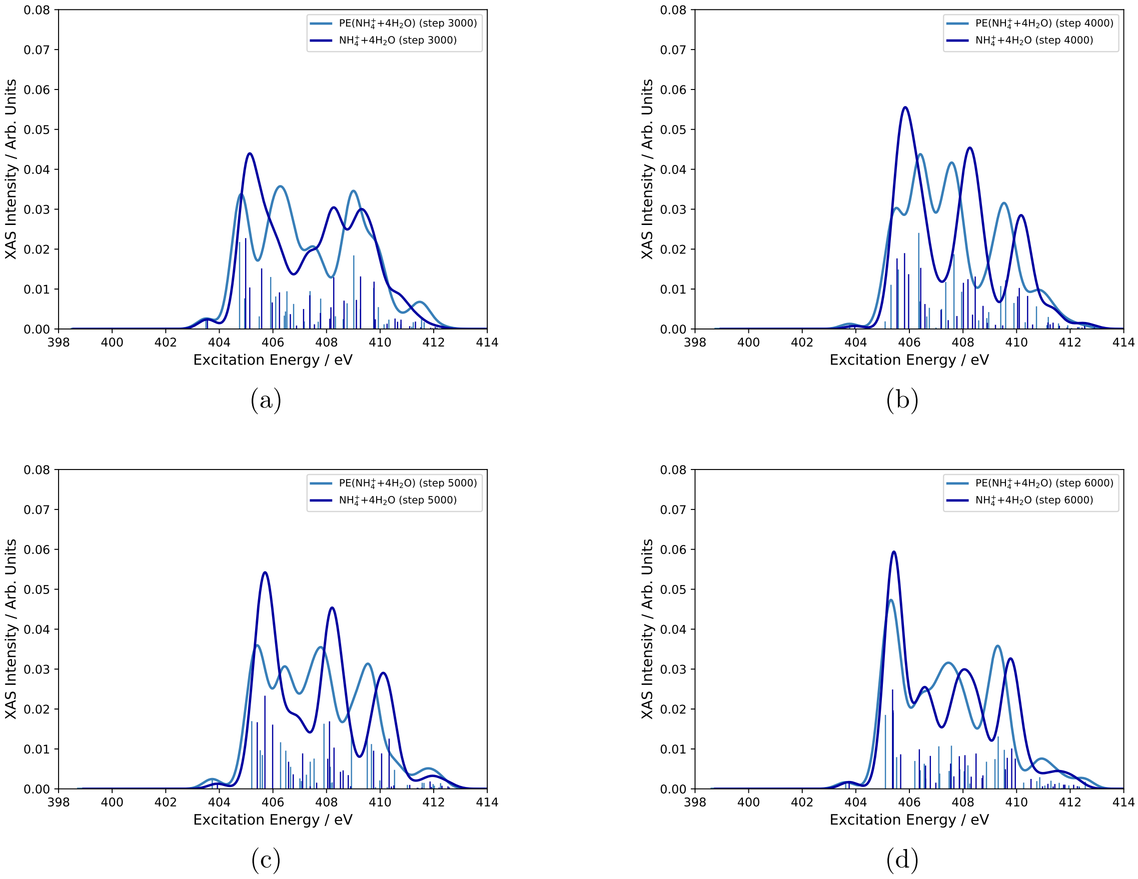

Figure S2: X-ray absorption spectra of ammonium at selected snapshots (3000-6000) surrounded by 4 water molecules with and without polarizable embedding (PE) at the CCSD/6-311++G** level of theory. The spectra have been shifted by -1.95 eV.

Effect of increased flexibility of the basis for the core orbitals

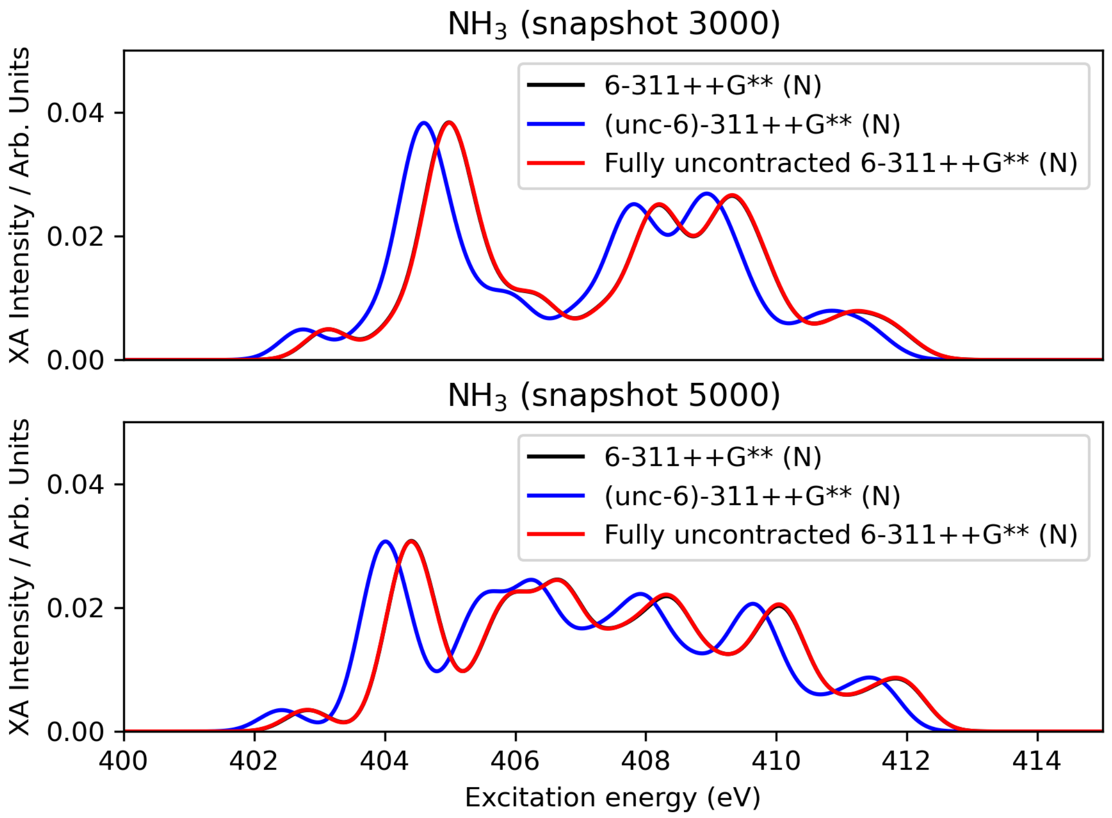

Figure S3: Effect of increasing the flexibility of the basis set to describe the core orbitals on the N atom. Regular 6-311++G** CCSD results are compared for one snapshot of each system with those obtained uncontracting the inner six orbitals as well as uncontracting all orbitals.

Choice of charges parametrization

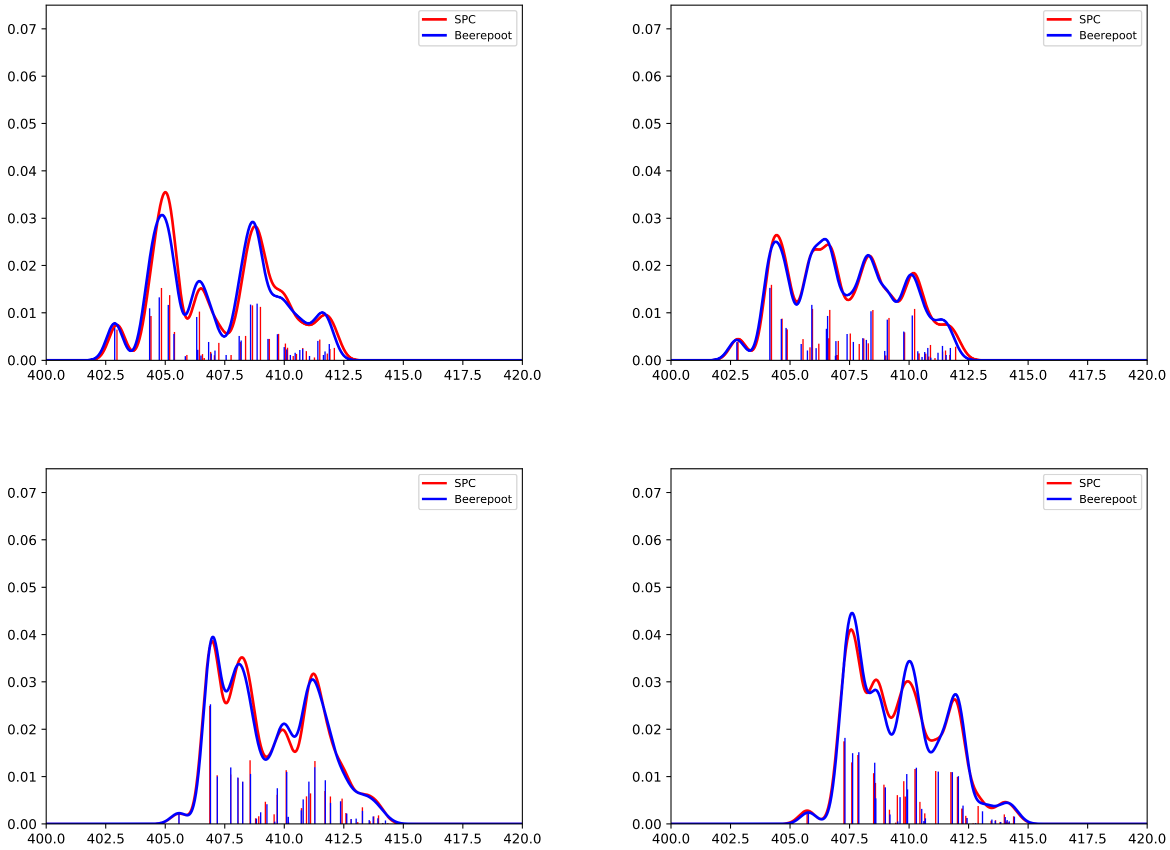

Figure S4: Comparison of different choices of charges in the PE description. The two upper panels are for two random snapshots (step 3000 and 5000, respectively) of the NH_3+4H_2O cluster; the two lower panels are for two random snapshots (step 3000 and 5000, respectively) of the NH_4^++4H_2O cluster.

Isotropic versus anisotropic polarizabilities

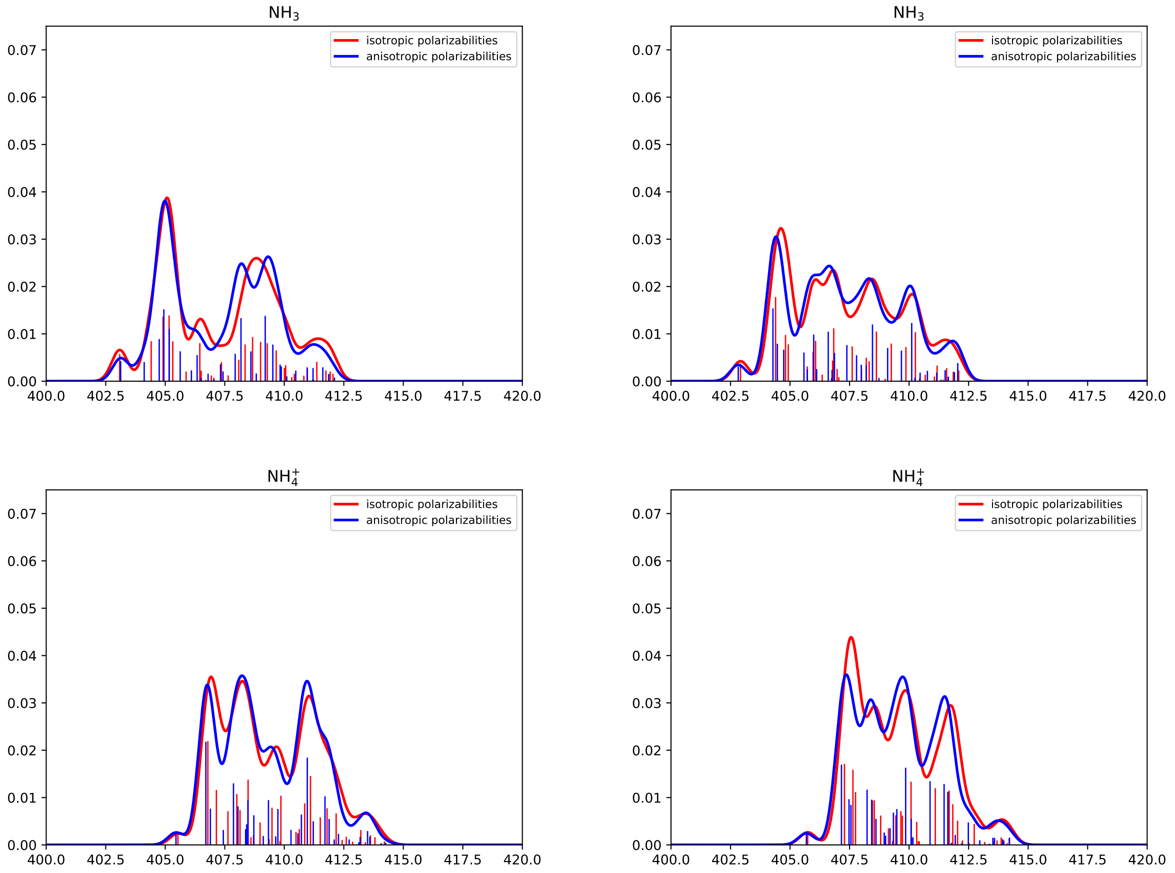

Figure S5: Comparison of different choices of polarizability in the PE description. The two upper panels are for two random snapshots (step 3000 and 5000, respectively) of the NH_3+4H_2O cluster; the two lower panels are for two random snapshots (step 3000 and 5000, respectively) of the NH_4^++4H_2O cluster.

Effective external field (EEF) effects

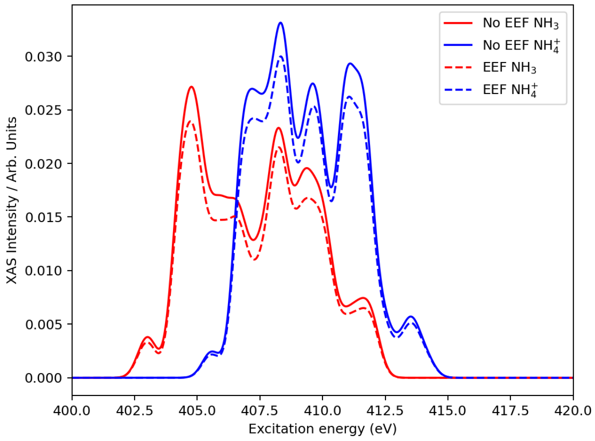

Figure S6: Comparison of the PE-CCSD/6-311++G** XA spectra of NH_3+4H_2O and NH_4^++4H_2O. The spectra are computed with/without inclusion of EEF effects.

Bulk-water effects

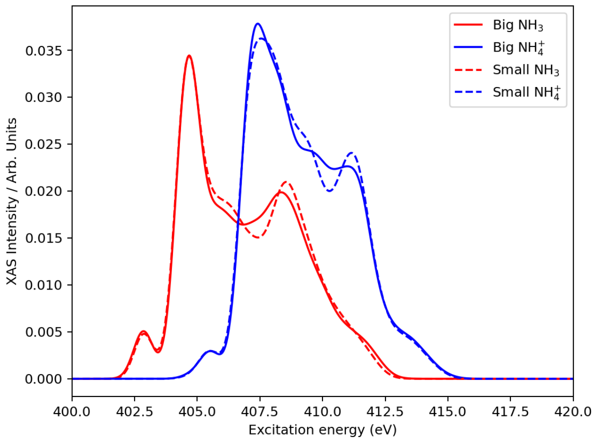

Figure S7: Effect of including larger environments in PE-CCSD/6-311++G** XA spectra calculations. The approach used in the main manuscript (Small) is compared to embeddings in which additional copies of the water in periodic images are included to mimic bulk-water effects (Big). The larger environments contain a QM region with ammonia/ammonium and 4 water molecules, an inner polarizable shell (12 Å) of loprop-parametrized waters, and an outer non-polarizable shell (25 Å) of non-polarizable SPC water. No EEF effects are included.

CC2 versus CCSD - two snapshots

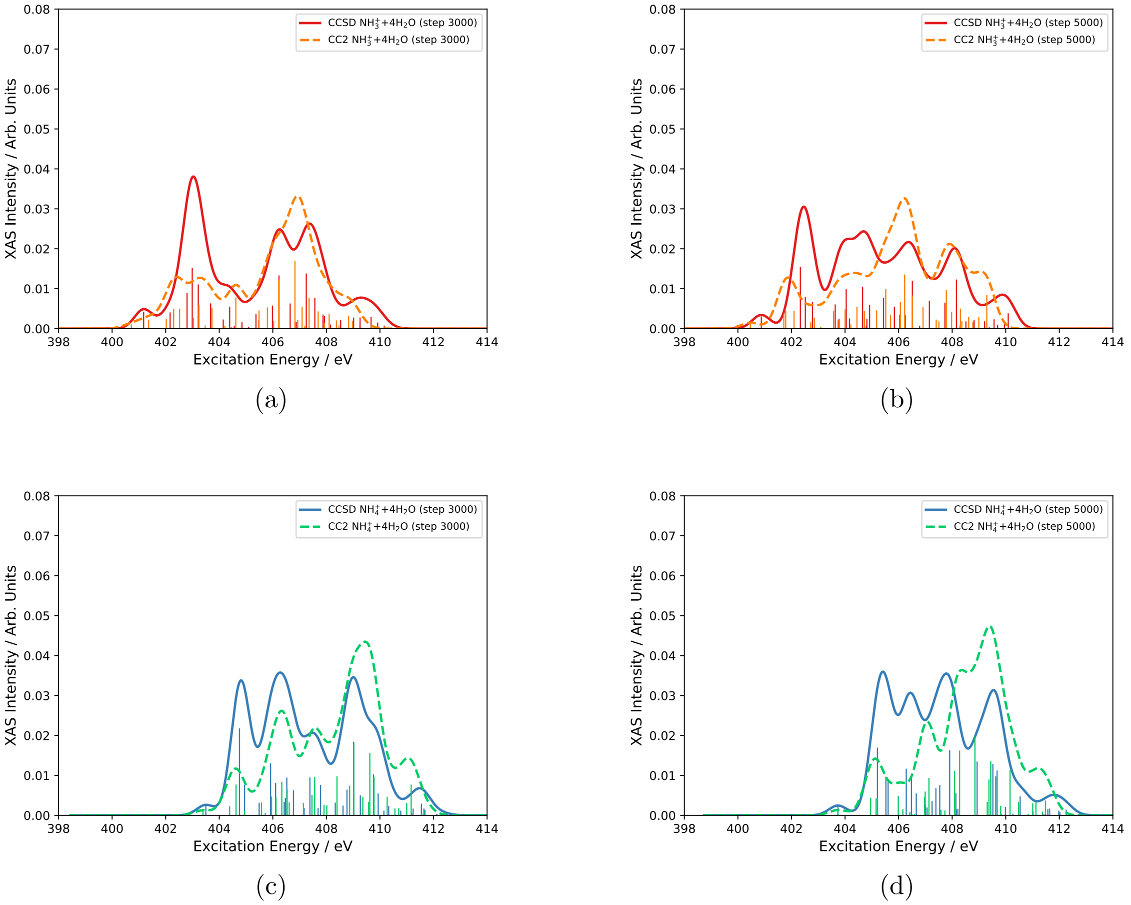

Figure S8: PE-CCSD and PE-CC2/6-311++G** X-ray absorption spectra of ammonia (upper panels) and ammonium (lower panels), both surrounded by 4 water molecules, at selected snapshots (3000 and 5000). The spectra have been shifted by −1.95 eV.

XAS for 4 representative structures

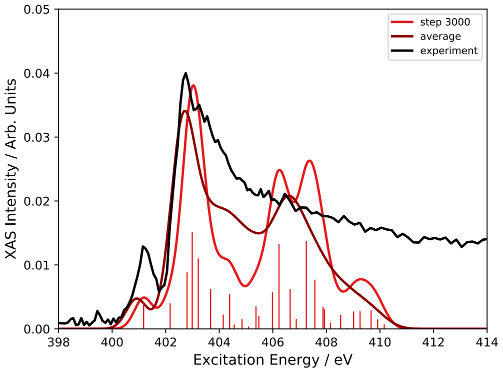

(a)

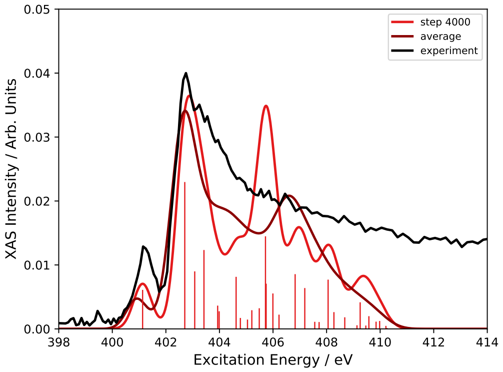

(b)

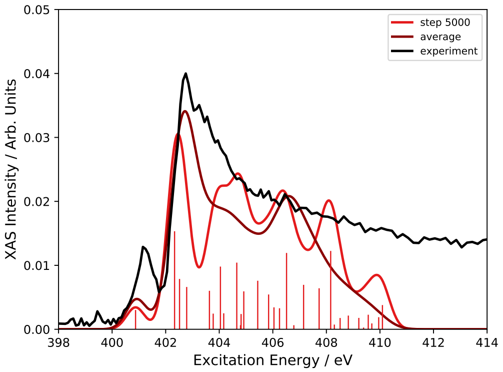

(c)

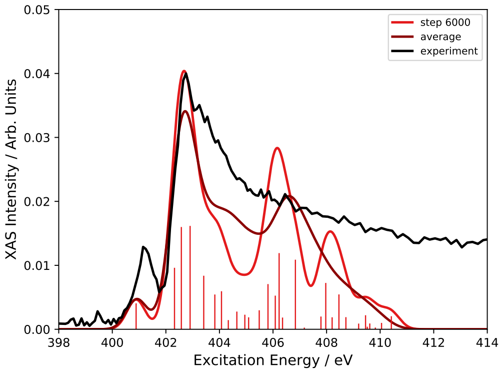

(d)

Figure S9: PE-CCSD/6-311++G** X-ray absorption spectra of NH_3 in water at selected (3000-6000) snapshots. The spectra have been shifted by -1.95 eV.

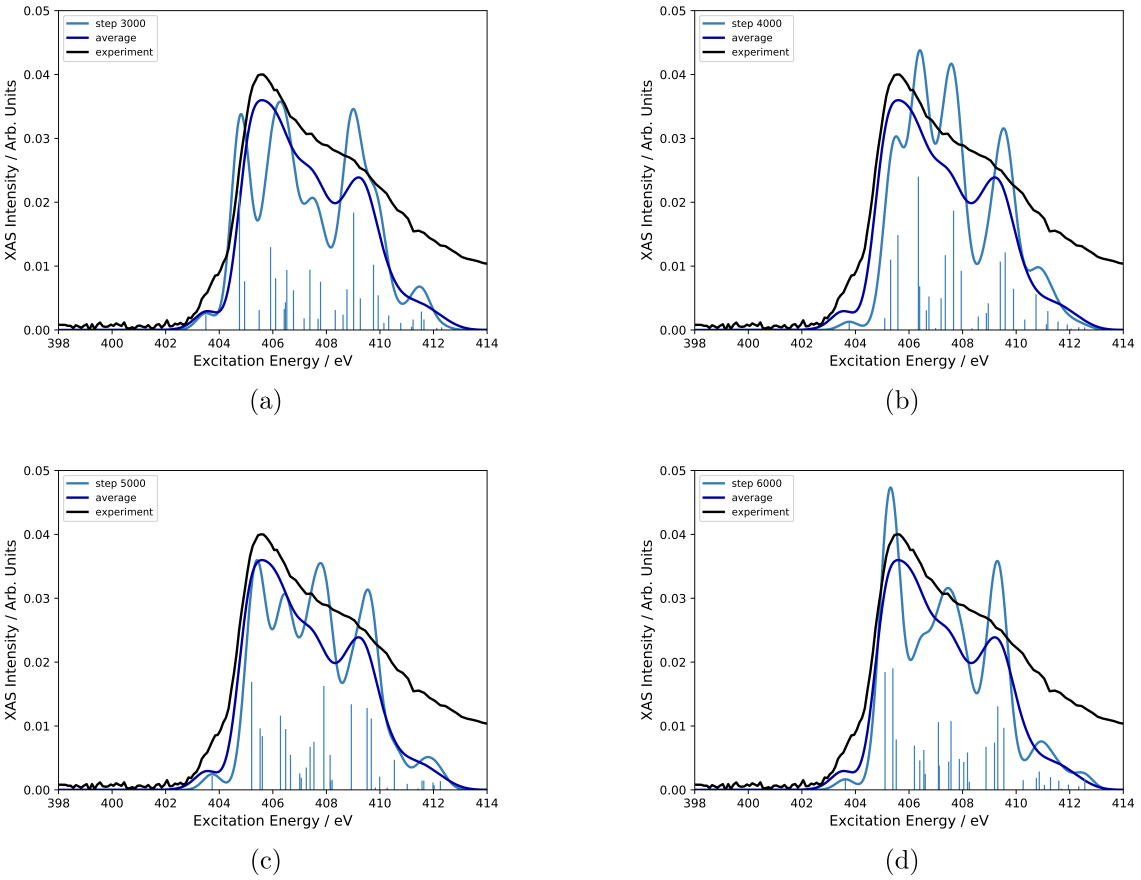

Figure S10: PE-CCSD/6-311++G** X-ray absorption spectra X-ray absorption spectra of ammonium in water at selected (3000-6000) snapshots. The spectra have been shifted by −1.95 eV.

Comparison of different TP-DFT methods, also enlarging the QM space

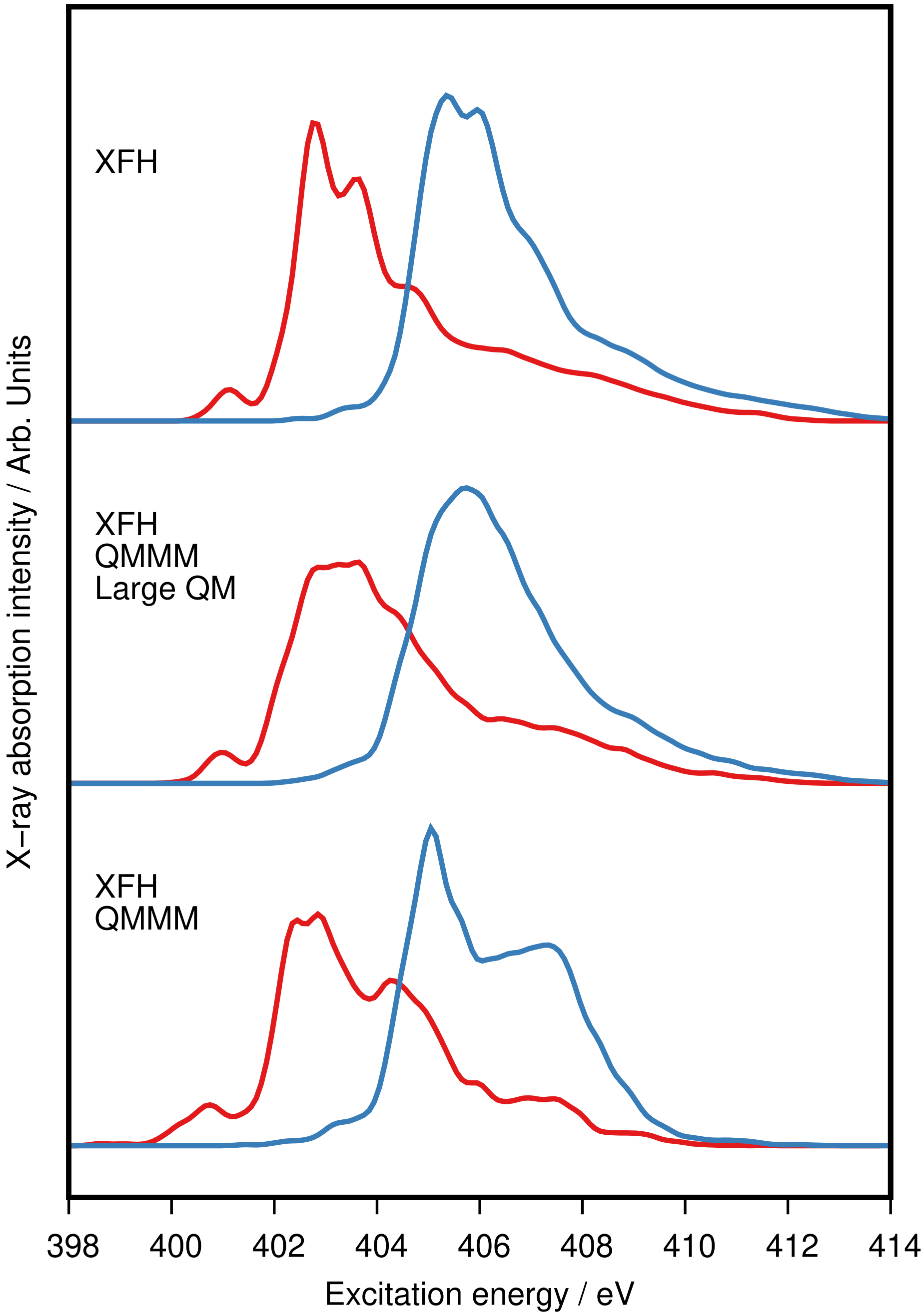

Figure S11: Comparison of the spectra obtained with different TP-DFT methods, and with the same choice of QMMM space (QMMM XFH) and enlarging the QM part to include all water molecules whose O atom is within 5 Å from the N atom (≈25 water molecules per snapshot).

Comparison PE-CCSD versus CPP-PE-TDDFT/CAM-B3LYP

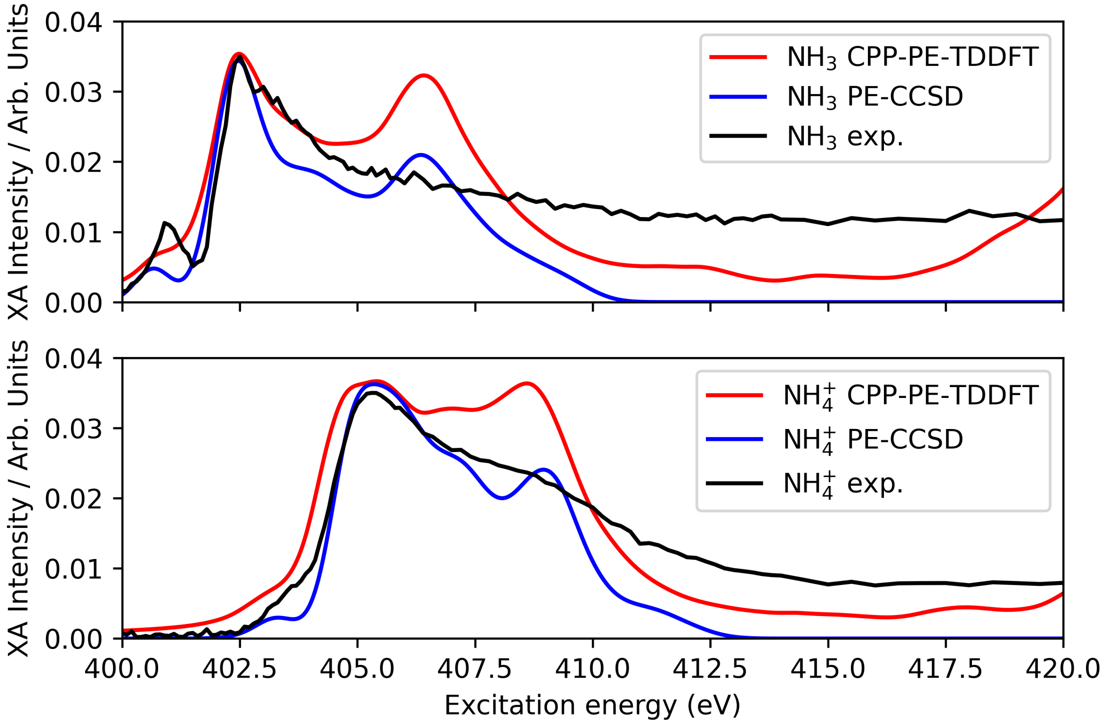

Figure S12: Comparison of the spectra obtained with PE-CCSD and with the PE complex-polarization-propagator time-dependent density functional method (CPP-PE-TDDFT) using the CAM-B3LYP functional. The TDDFT spectra have been shifted by 14.5 eV.

Acknowledgement

The authors thank the COST EuSpec action. M.L.V. and S.C. thank N.H. List for discussions. S.C. and M.L.V. acknowledge financial support from DTU Chemistry (Ph.D. start-up grant) and the Independent Research Fund Denmark, DFF-RP2 grant no. 7014-00258B. S.C. and J.K. acknowledge the European Union's Horizon 2020 research and innovation programme under the Marie Skłodowska-Curie grant agreement No 765739, "COSINE–European Training Network on Computational Spectroscopy In Natural sciences and Engineering". M.O. acknowledges support from the European Union's Horizon 2020 research and innovation programme under the Marie Skłodowska-Curie grant agreement No 860553 and from the Carl Tryggers Foundation (contract CTS18:285). Part of the calculations were enabled by resources provided by the Swedish National Infrastructure for Computing (SNIC) at at the Swedish National Supercomputer Center (NSC), the High Performance Computer Center North (HPC2N), and Chalmers Centre for Computational Science and Engineering (C3SE) partially funded by the Swedish Research Council through grant agreement no. 2018-05973. We acknowledge Maria Ekimova, Wilson Quevedo, Philippe Wernet and Erik T. J. Nibbering, whose experimental data (reproduced from Ref. 1) are used as a reference for our current calculations.

References

(1) Ekimova, M.; Quevedo, W.; Szyc, Ł.; Iannuzzi, M.; Wernet, P.; Odelius, M.; Nibbering, E. T. J. Aqueous Solvation of Ammonia and Ammonium: Probing Hydrogen Bond Motifs with FT-IR and Soft X-ray Spectroscopy. *J. Am. Chem. Soc.* **2017**, *139*, 12773–12783.
